# Supplementary material for: Cell state-specific cytoplasmic density controls spindle architecture and scaling
Source: Nat Cell Biol. 2025 Jun 13;27(6):959–71. doi: 10.1038/s41556-025-01678-x (PMC12173940; doi:10.1038/s41556-025-01678-x)

nature cell biology

Supplementary information https://doi.org/10.1038/s41556-025-01678-x

Cell state-specific cytoplasmic density controls spindle architecture and scaling

In the format provided by the authors and unedited

Supplementary Note

1. Theoretical model

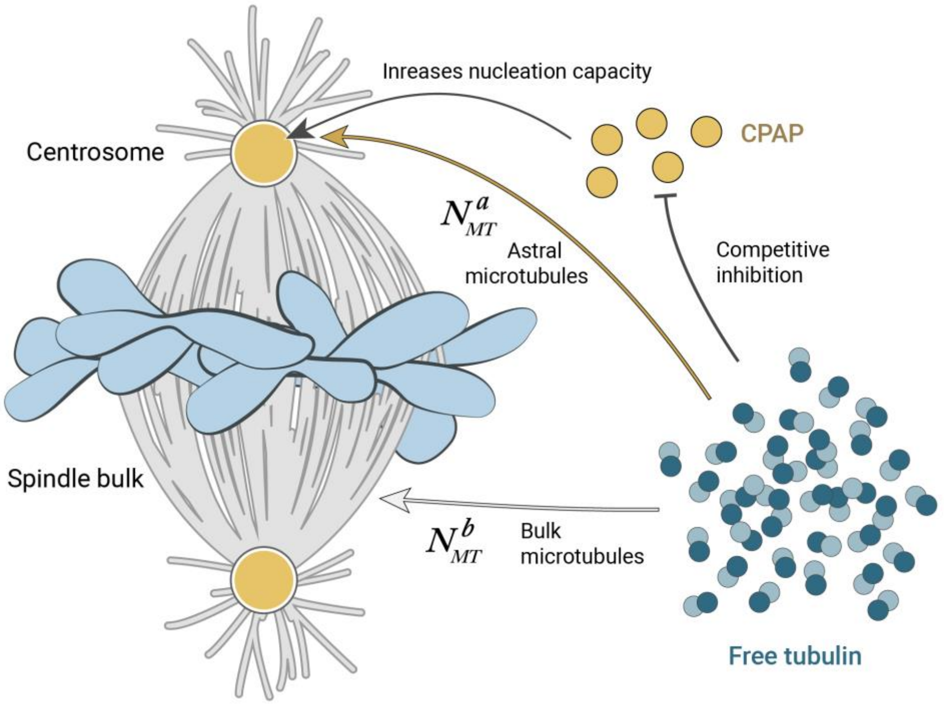

Supplementary Note Figure 1: Schematic explaining the redistribution of mitotic microtubule populations via the inhibition of CPAP by free tubulin.

In this study, we have shown that differentiating cells change their spindle architecture and size by redistributing microtubule growth to the spindle poles at the expense of the spindle bulk (Suppl. Note Fig. 1). While the total microtubule number scales with cell volume (Extended Data Fig. 3a iv), the distribution into either astral or spindle bulk microtubules defines final spindle size. Thus, we propose a scaling of astral microtubule number with cell volume based on the mechanism suggested by Rieckhoff and colleagues [1]: astral microtubule number has a Michaelis-Menten dependence on cell volume and is proportional to the number of active (centrosomal) nucleators in the cell:

$$N^a_{MT} = \sigma\, n^{free}_{CPAP}\, \frac{V}{1 + V/V_0} \qquad (1)$$

where N^a_{MT} denotes the number of astral microtubules, V the cell volume and n^{free}_{CPAP} the concentration of free CPAP molecules (analogous to an active nucleator in [1]). The model

parameters are σ and V_0, which respectively denote the proportionality constant between the number of astral microtubules and the free CPAP concentration, and the reaction-diffusion volume of CPAP. The reaction-diffusion volume is defined as the volume in which the reaction is not limited by diffusion. The observed inhibition of CPAP by free tubulin (Fig. 6) is an important aspect of the system, which we incorporate into the theory in the following way: tubulin competitively inhibits CPAP through a simple binding reaction with a dissociation constant κ_c. CPAP bound to tubulin is unable to increase centrosomal nucleation capacity, only free CPAP molecules can, their concentration being:

$$n_{CPAP}^{free} = n_{CPAP}\left(1 + n_T^{free}/\kappa_c\right)^{-1} \quad (2)$$

where n_{CPAP} denotes the total concentration of CPAP in the cell (free + bound to tubulin) and n_T^{free} the soluble tubulin concentration in the cell. Tubulin is either soluble or polymerized, which can be expressed with the following equation:

$$n_T^{free} = n_T - \frac{N_{MT}\,\lambda}{V} \quad (3)$$

where n_T denotes the total tubulin concentration in the cell and $N_{MT} = N_{MT}^a + N_{MT}^b$ the total number of microtubules, which is the sum of the number of astral microtubules N_{MT}^a with the number of spindle bulk microtubules N_{MT}^b. Here we also combine the linear density of tubulin α, the average growth velocity v_{MT} and the average lifetime of a microtubule τ_{MT} in a single parameter λ ($\lambda = \alpha\, v_{MT}\, \tau_{MT}$), which represents the number of tubulin molecules found in an average microtubule and which we assume to not significantly change between undifferentiated and differentiated cells based on our experimental evidence (Fig. 2e and Extended Data Fig. 3a i, ii).

To test whether our model can correctly predict a higher number of astral microtubules N_{MT}^a for differentiated cells, we connect both microtubule populations, i.e. the number of microtubules in the spindle bulk N_{MT}^b and astral microtubules N_{MT}^a, using our experimentally determined ratio f_T (Fig. 2i):

$$N_{MT}^{a} = N_{MT}^{b}\, f_T\, . \tag{4}$$

Thus, our model is fully described by equations (1)-(4) and these already qualitatively predict more astral microtubules and less bulk microtubules in differentiated cells when compared to undifferentiated cells with the same cell volume V.

2. Model predictions

First, we have to establish which quantities of the model stay constant and which change when we compare undifferentiated and differentiated cells. As previously mentioned, our experimental results show that we can consider microtubule dynamics related quantities such as λ to be the same. The dissociation constant κ_c between tubulin and CPAP has been reported to be 3.6 µM [2] and we assume it to have this value for both undifferentiated and differentiated cells. Lastly, we assume the proportionality constant σ between the number of astral microtubules N_{MT}^{a} and the concentration of free CPAP n_{CPAP}^{free} to be the same for both systems. This assumption can be justified by the observation that in both cell states an increase in centrosome size correlates with nucleation capacity (Figs. 2k and 3c).

Quantities that we can expect to change across differentiation are those related to dry mass density, given that we observe an approximately 10% reduction of dry mass density in differentiated cells compared to undifferentiated cells (Fig. 5b). In our model, the total concentration of CPAP n_{CPAP} and the total concentration of tubulin n_T are both reduced in differentiated cells by the same factor as the dry mass density (see Fig. 4, Fig. 5c and Suppl. Note Fig. 2). Finally, we can expect the reaction-diffusion volume of CPAP V_0 to be larger for differentiated cells because of their lower dry mass density, which decreases crowding. With these considerations, we see from equation (3) that the free tubulin concentration n_T^{free} is lower in differentiated cells than undifferentiated cells of the same volume. Starting from

equation (2), let us derive under which conditions the free CPAP concentration n_{CPAP}^{free} is higher in differentiated cells. The expressions for both cell states are:

$$n_{CPAP,P}^{free} = n_{CPAP} \left(1 + \frac{n_T - N_{MT}\,\lambda/V}{\kappa_c}\right)^{-1}$$

$$n_{CPAP,D}^{free} = q\, n_{CPAP} \left(1 + \frac{q\, n_T - N_{MT}\,\lambda/V}{\kappa_c}\right)^{-1}$$

where the subindices *P, D* denote undifferentiated (pluripotent) and differentiated quantities, respectively. The factor $0 < q < 1$ describes the decrease of the overall mass density in differentiated cells compared to undifferentiated cells. If we consider cells of the same volume, then the term $N_{MT}\,\lambda/V$ is the same for both cell states. We can rearrange the expression for $n_{CPAP,D}^{free}$ in the following way:

$$n_{CPAP,D}^{free} = n_{CPAP} \left[1 + \frac{n_T - N_{MT}\,\lambda/V}{\kappa_c} + \left(\frac{1}{q} - 1\right)\left(1 - \frac{N_{MT}\,\lambda}{V\,\kappa_c}\right)\right]^{-1}$$

From the equation above we can see that the condition for $n_{CPAP,D}^{free} > n_{CPAP,P}^{free}$ is:

$$\frac{N_{MT}\,\lambda}{V\,\kappa_c} = \frac{n_T^{pol}}{\kappa_c} > 1$$

where we defined $n_T^{pol} = \frac{N_{MT}\,\lambda}{V}$ as the effective concentration of polymerised tubulin in the cell.

Based on our measurements of total cellular tubulin (Extended Data Table 1), we estimate 50% of the total amount of tubulin to be in polymer form $n_T^{pol} \simeq 6$ µM, both in astral and spindle microtubules. This is consistent with previous reports [3].

As mentioned above, the dissociation constant $\kappa_c = 3.6$ µM [2], so we indeed have $\frac{n_T^{pol}}{\kappa_c} \simeq 1.7 > 1$. Therefore, by considering equation (1), we can see that qualitatively both the higher values

of n_{CPAP}^{free} and V_0 in differentiated cells will result in a higher number of astral microtubules N_{MT}^{a} .

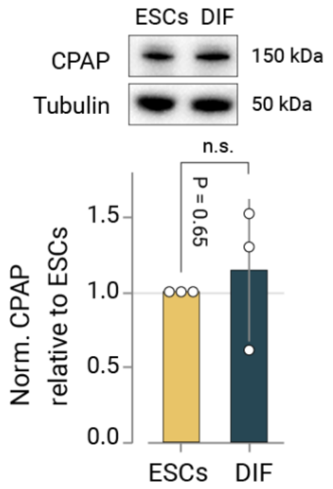

Supplementary Note Figure 2: CPAP:total tubulin stoichiometry is unchanged between the differentiation states.

Cellular levels of CPAP after 48 h of differentiation (DIF) relative to the undifferentiated ESCs, probed by western blotting and normalised to tubulin (N = 3). Bars show the mean, errors show the standard deviation. Significance tested by Welch's t-test (two-sided), n.s.: $P > 0.05$.

3. Model fitting

Now that we have shown that the model works qualitatively, we next want to connect it to our main measured quantities. To estimate the number of microtubules in the spindle bulk, we link our experimentally well-defined spindle volumes V_S and tubulin concentration in the spindle bulk n_T^b by

$$N_{MT}^{b} = \frac{V_S}{n_T^b \, \lambda} \; . \tag{5}$$

Once we determined N_{MT}^{b} , we can obtain N_{MT}^{a} using equation (4). Note that we assume n_T^b to be the same for undifferentiated and differentiated cells (based on Extended Data Figures 2j, 3c and 4).

To fit our data with the model, we use equations (4) and (5) to estimate N_{MT}^{a} with our data for V_S and rewrite equation (1) as follows:

$$N_{MT}^{a} = \sigma\, n_{CPAP}\, \frac{1}{1+\chi} \frac{V}{1+V/V_0}\,, \tag{6}$$

where $\chi = \frac{n_T^{free}}{\kappa_c}$ and V_0 are treated as fit parameters. Next, we have to estimate the parameter σ, which collapses the influence of CPAP on centrosome size and nucleation capacity. To do so, we rearrange equation (1):

$$\sigma = \frac{N_{MT}^{a}\left(1+n_T^{free}/\kappa_c\right)(1+V/V_0)}{n_{CPAP}\, V}\,. \tag{7}$$

To estimate N_{MT}^{a} in the above equation, we can use experimentally measured spindle quantities:

$$N_{MT}^{a} = f_T\, \frac{V_S^b\, n_T^b}{\lambda}\,. \tag{8}$$

4. Parameter values and estimates

All parameter values are given in Extended Data Table 1, more specifically the column that corresponds to cells binned by volume (V_{bin}). For the purpose of estimating σ using equation (7), we need an estimate for V_0, the reaction diffusion volume, and for $\chi = n_T^{free}/\kappa_c$. For these, we assign the following values: $V_0 = 3000$ µm^3, which is consistent with our data (Fig. 2k) and both cell states being in the linear scaling regime [1,4,5]. As described above, we estimated $n_T^{free}/\kappa_c \simeq 1.7$ based on our measurements and a published κ_c [2]. Plugging these values into equations (7) and (8) results in a value range $\sigma = 0.003 \sim 0.006$. Therefore, we assume $\sigma = 0.0045$ for both undifferentiated and differentiated cells in the following fits of the experimental data to the theory.

5. Model results & discussion

To test our model, we take our estimates of N_{MT}^{a} for both cell states, bin them by cell volume analogous to the experimental data, and fit the respective averages of N_{MT}^{a} to equation (6). Supplementary Note Fig. 3a shows the binned averages for N_{MT}^{a}, the respective fit curves, and the obtained fit parameters for both cell states. For differentiated cells, the fitted value of V_0 is larger than for undifferentiated cells but lies in the same order of magnitude ($\sim 10^3$ μm^3). This is consistent with a drop in cellular mass density and further implies that the number of astral microtubules will start to saturate with cell volumes $\gtrsim 10^3$ μm^3. This is indeed what we observe for astral microtubules (Fig. 2k) and centrosome volume (Fig. 3c, Extended Data Fig. 6). For the fit parameter $\chi = \frac{n_T^{free}}{\kappa_c}$ we can see that differentiated cells exhibit a lower value than undifferentiated cells. This again is consistent with a reduced free tubulin concentration in differentiated cells. Importantly, the model gives a reasonable fit to the binned cell volumes as well as the respective point cloud of our experimental data (as presented in the inset plot of Supplementary Note Fig. 3a).

In Supplementary Note Fig. 3b, we test how the fit parameters V_0 and χ influence the number of astral microtubules as a function of cell size as defined by equation (6). Changing the parameter χ results in the same curve multiplied by a numerical prefactor. This is consistent with our biochemical perturbation experiments (see Fig. 6i), where we inhibit the CPAP tubulin interaction, which would decrease χ. The parameter V_0 represents the cell volume at which the system reaches half of the saturation value, but it also affects the saturation value itself as we can see by considering equation (6) in the limit $V/V_0 \gg 1$:

$$N_{MT}^{a} = \sigma\, n_{CPAP}\, \frac{1}{1 + \chi}\, V_0.$$

This is consistent with the number of astral microtubules as well as centrosome volumes reaching the saturation regime at different levels for both cell states (Figs. 2k and 3c).

A main point in our observation is that the total number of microtubules scales with cell volume and does not change between cell states (Extended Data Fig. 3a vi, c). To test

whether our theory would correctly reproduce this observation, we lastly estimate the total number of microtubules. Indeed, we find the total number of microtubules to scale with cell volume and to not differ significantly between cell states (Suppl. Note Fig. 3c).

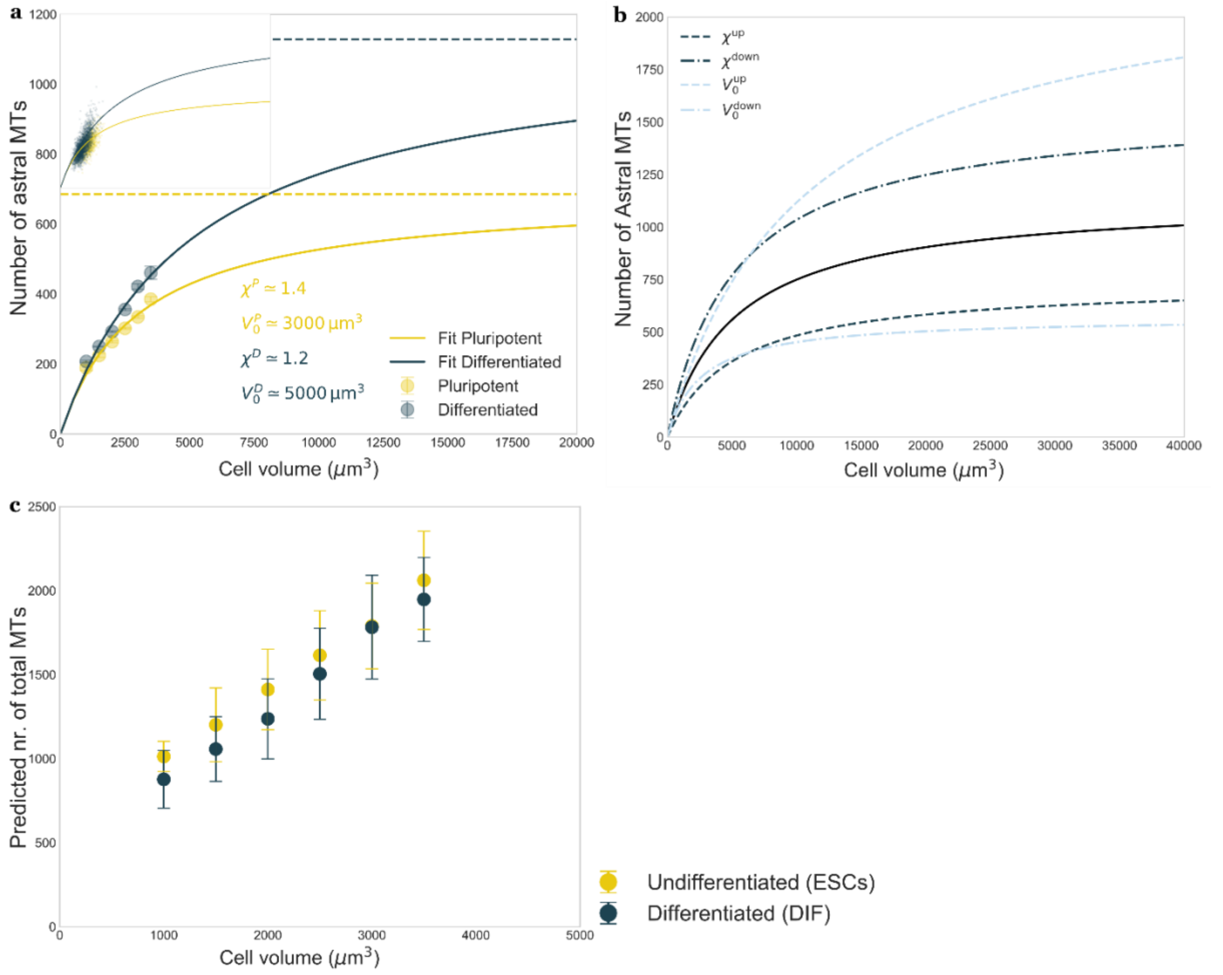

Supplementary Note Figure 3: Results of the theoretical model.

a) Fit of the theory to experimental data. For both cell states (undifferentiated: yellow; differentiated: blue), the number of astral microtubules is estimated from the spindle volume data and grouped by cell volume in intervals of 500 μm³. The large circles depict the averages for each cell volume interval and are fitted to equation (6), the error bars show the standard deviation. Solid curves represent the fit curves, obtained from the resulting fit parameters (depicted in the plot). Dashed lines depict the saturation value. Inset plot shows the data cloud with the same fit curves.

b) Influence of the fit parameters on the theoretical curves. The solid black curve depicts the fit for differentiated cells (as in a) and the colored curves are obtained by increasing or decreasing one of the two fit parameters by a factor of two.

c) Theory predicts similar total numbers of microtubules for both cell states. The total number of microtubules is estimated from the spindle volume data and grouped by cell volume in intervals of 500 μm³. The large circles depict the averages for each cell volume interval, the error bars show the standard deviation.

As all theoretical models, our model likely makes significant over-simplifications. However, our theory is in line with former quantitative descriptions of spindle scaling [1,6,7]. Firstly, in [1], nucleators are active in the cytoplasm and inactive when sequestered to the cell membrane, which allows for embryonic scaling. In our version, we treat CPAP as the nucleator of astral microtubules, which might be direct or indirect, and here, the activity of CPAP is regulated by free tubulin, which is coupled to cell state. This hints towards a similar regulation via nucleation inhibition: in the embryonic context physical sequestration by membrane, and in the differentiation context, biochemical inhibition by tubulin where equally sized cells (with identical cell surface-to-volume ratio) show a significant difference in spindle size.

Secondly, in our model the amount of polymerised tubulin is conserved based on cell volume and we relate microtubule number to spindle volume as in [6]. In the theory, the values assumed for the concentration of polymerised tubulin in the spindle bulk n_T^b are taken from our measurements, which cannot discern which fraction of bulk tubulin is free and polymerised. However, our measurements of microtubule growth velocities and EB1 comet density within the spindle (see Fig. 2e and Extended Data Fig. 3c) imply the ratio between free and polymerised tubulin in the spindle bulk to not significantly differ between cell states. Thus, a potential overestimate of polymerised microtubule numbers would affect both cell states and not translate into a different scaling behaviour.

The dilution of the cytoplasm observed in our differentiated cells (Fig. 5a, b) could have significant effects on the diffusion and reaction rates of important macromolecules such as tubulin and CPAP. In fact, a recent study [8] reported that microtubule dynamics in *S. pombe* yeast cells were affected by osmotic challenges. However, the *S. pombe* cells have a much higher cytoplasmic density (~ 200 mg/mL) than the mouse embryonic stem cells studied here (~ 120 mg/mL), so the effects of crowding can be expected to be stronger in *S. pombe* cells. In our system, however, we do not observe any changes in the growth speed of microtubules between differentiated and undifferentiated cells (Fig. 2e and Extended Data Fig. 3a i, ii). Moreover, tubulin in both cell types is well within its reaction-diffusion volume as evidenced by the scaling of spindle volume with cell volume (Fig. 1h, i). Therefore, we do not expect

tubulin diffusion to be limiting for spindle assembly reactions and the effects of dilution are captured by the different protein concentrations. Finally, our model does account for possible changes in the diffusion and reaction rates of CPAP in both cell types as evidenced by the different fitted values of the parameter V_0 (Suppl. Note Fig. 3a), which represents the reaction-diffusion volume of CPAP.

What distinguishes our model from previous established ones [1,6,7] is that it proposes a regulatory mechanism for the partitioning of polymerised tubulin into either spindle bulk or astral microtubules while scaling with cell volume.

References

1. Rieckhoff, E. M. *et al.* Spindle scaling is governed by cell boundary regulation of microtubule nucleation. *Curr. Biol.* **30**, 4973-4983.e10 (2020).
2. Mariappan, A. *et al.* Inhibition of CPAP-tubulin interaction prevents proliferation of centrosome-amplified cancer cells. *EMBO J.* **38**, e99876 (2019).
3. Zhai, Y., Kronebusch, P. J., Simon, P. M. & Borisy, G. G. Microtubule dynamics at the G2/M transition: abrupt breakdown of cytoplasmic microtubules at nuclear envelope breakdown and implications for spindle morphogenesis. *J. Cell Biol.* **135**, 201–214 (1996).
4. Courtois, A., Schuh, M., Ellenberg, J. & Hiiragi, T. The transition from meiotic to mitotic spindle assembly is gradual during early mammalian development. *J. Cell Biol.* **198**, 357–370 (2012).
5. Crowder, M. E. *et al.* A comparative analysis of spindle morphometrics across metazoans. *Curr. Biol.* **25**, 1542–1550 (2015).
6. Good, M. C., Vahey, M. D., Skandarajah, A., Fletcher, D. A. & Heald, R. Cytoplasmic volume modulates spindle size during embryogenesis. *Science* **342**, 856–860 (2013).
7. Reber, S. B. *et al.* XMAP215 activity sets spindle length by controlling the total mass of spindle microtubules. *Nat. Cell Biol.* **15**, 1116–1122 (2013).
8. Molines, A. T. *et al.* Physical properties of the cytoplasm modulate the rates of microtubule polymerization and depolymerization. *Dev. Cell* **57**, 466-479.e6 (2022).

Supplementary Note Figure 2 - Uncropped Blots

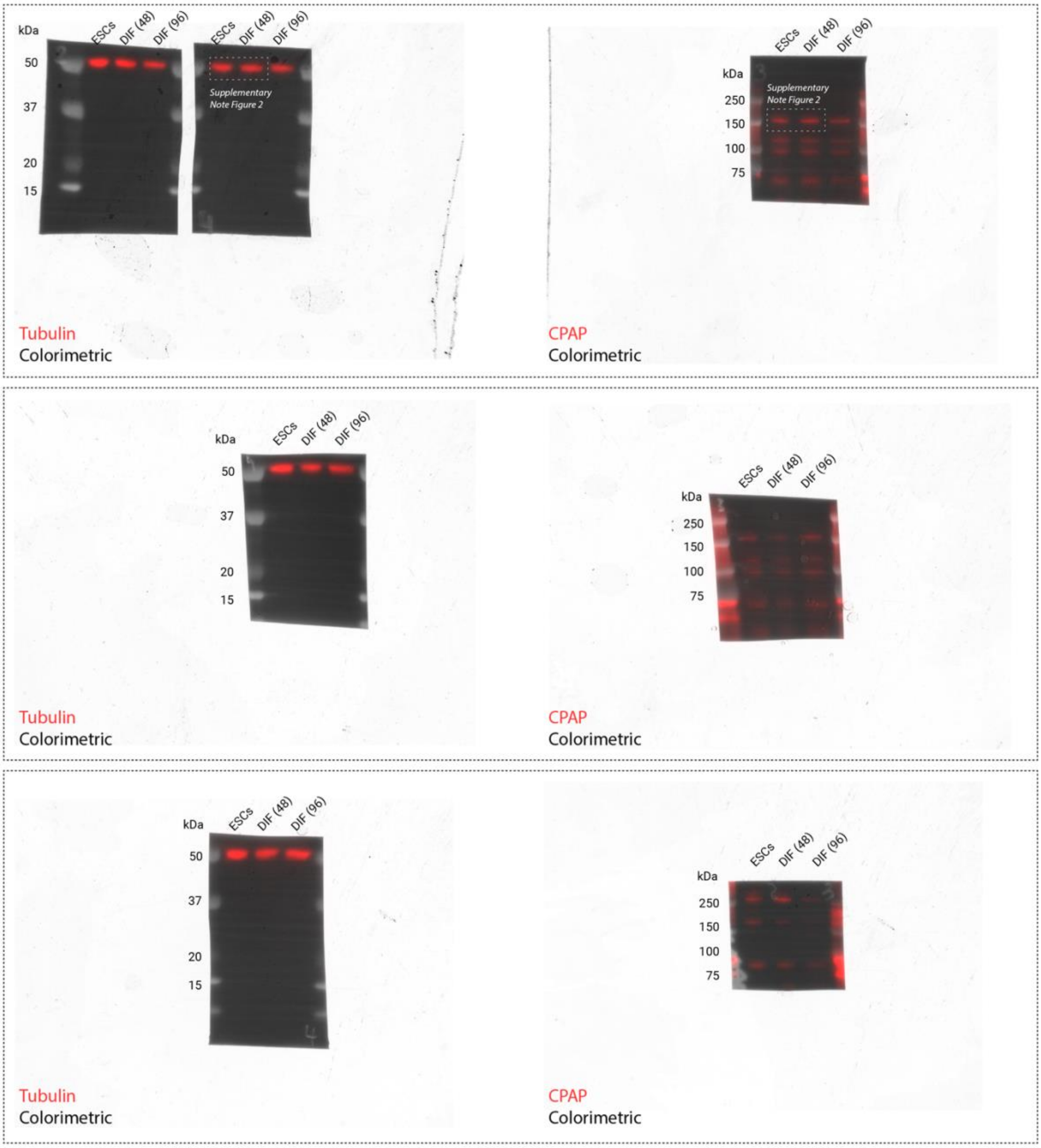

Supplement: Supplementary file 1 — Supplementary Note and Figs. 1–3. [file 41556_2025_1678_MOESM1_ESM.pdf]
