# Supplementary material for: Cell state-specific cytoplasmic density controls spindle architecture and scaling
Source: Nat Cell Biol. 2025 Jun 13;27(6):959–71. doi: 10.1038/s41556-025-01678-x (PMC12173940; doi:10.1038/s41556-025-01678-x)
Supplement: Supplementary file 8 — Supplementary Table 1: Key measurements of cell and spindle morphometrics. Overview summarizing key measurements (mean ± s.d.) and calculations in undifferentiated ESCs (ESCs) versus early-differentiated cells (DIF), as well as in cells during hypo-osmotic treatment, or treatment with the small molecule inhibitor CCB02. Vbin compares ESCs versus DIF with comparable cell volumes (2,000–2,500 µm3). Iso, isotonic control; DMSO, control for CCB02; ND, not determined; γTub, γ-tubulin. Supplementary Table 2: Summary of the microscope settings of the imaging tasks that were used to run the adaptive feedback microscopy pipeline. [file 41556_2025_1678_MOESM8_ESM.pdf]

	ESCs (0 h)	DIF (48 h)	V_{bin} (ESCs/DIF)	Hypo-osmotic	CCB02
**Cell volume V (µm³)**	2870 ± 302	2560 ± 335	2277 ± 141/<br>2277 ± 144	3320 ±<br>(Iso: 3085)	2926 ± 412
**Spindle volume V_s (µm³)**	363 ± 65	307 ± 67	305 ± 52/<br>251 ± 48	388 ± 64<br>(Iso: 424)	328 ± 67
**% of total tubulin in spindle**	46.3 ± 3.8	43.9 ± 5.0	46.3 ± 4.6/<br>42.4 ± 4.7	n.d.	n.d.
**Centrosome volume (µm³)**	4.7 ± 1.3	4.7 ± 1.1	4.0 ± 1.0/<br>4.6 ± 1.0	6.0 ± 1.7<br>(Iso: 5.4)	6.4 ± 1.2<br>(DMSO: 6.2, Dif: 6.4)
**% of total γTub in centrosomes**	2.9 ± 1.4	3.6 ± 1.9	3.1 ± 1.8/<br>3.6 ± 1.9	2.5 ± 1.0<br>(Iso: 2)	3.4 ± 1.5<br>(DMSO: 2.9)
**% of total γTub on spindle (excluding centrosomes)**	21.5 ± 10.0	21.5 ± 11.7	22.4 ± 12.7/<br>21.4 ± 12.1	15.1 ± 2.7<br>(Iso: 16.2)	n.d.
**MT growth speed v_{MT} (µm/s)**	0.26 ± 0.03	0.26 ± 0.03	0.26 ± 0.02/<br>0.25 ± 0.04	n.d.	n.d.
**Total number of growing MTs (EB1)**	66 ± 14	57 ± 11	61 ± 15/<br>54 ± 11	n.d.	n.d.
**Number of bulk MTs N_{MT}^{b} (EB1)**	54 ± 12	43 ± 8	51 ± 13/<br>41 ± 9	n.d.	n.d.
**Number of astral MTs N_{MT}^{a} (EB1)**	12 ± 4	13 ± 4	10 ± 3/<br>13 ± 3	n.d.	n.d.
N_{MT}^{a} **:** N_{MT}^{b} **(EB1)**	0.23 ± 0.05	0.31 ± 0.09	0.19 ± 0.04/<br>0.33 ± 0.08	n.d.	n.d.
**Number of astral MTs (fixed & projected)**	14 ± 3	17 ± 3	9.2 ± 3.2 /<br>16.9 ± 2.9	32.2 ± 5.4<br>(Iso: 25.4)	25.6 ± 8.5<br>(DMSO: 21.7)
**$\alpha\beta$-tubulin mass (% cell protein)**	1.56 ± 0.08	1.51 ± 0.09	n.d.	n.d.	n.d.
**Cell mass density ρ (mg/ml)**	140 ± 12	125 ± 11	136 ± 10/<br>124 ± 11	112 ± 9<br>(Iso:118)	n.d.
**Cell dry mass (pg)**	291 ± 48	257 ± 52	304 ± 36/<br>278 ± 29	271 ± 38<br>(Iso 274)	n.d.
**Cell tubulin concentration (µM) (assuming protein dry mass = 0.6 * cell dry mass)**	13.1	11.4	n.d.	n.d.	n.d.

**Spindle bulk tubulin conc. (incl. centrosomes) (µM)**	48.0	41.6	48.0 ± 4.8 / 43.0 ± 4.6<br><br>Used in model: 45.5	n.d.	n.d.
**Microtubule lifetime τ (s)**			18.2		
**Linear tubulin density on a microtubule α (µm^{-1})**			1625		
**Dissociation constant between tubulin and CPAP κ_C (µM)**			3.7		
**CPAP concentration in the cell (nM)**			200 / 180		

Supplementary References

Brugués, J., Nuzzo, V., Mazur, E. & Needleman, D. J. Nucleation and transport organize microtubules in metaphase spindles.
Cell 149, 554–564 (2012).

Needleman, D. J. et al. Fast microtubule dynamics in meiotic spindles measured by single molecule imaging: evidence that the spindle environment does not stabilize microtubules. Mol. Biol. Cell 21, 323–333 (2010).

Goodson, H. V. & Jonasson, E. M. Microtubules and microtubule-associated proteins. Cold Spring Harb. Perspect. Biol. 10, a
022608 (2018).

Sharma, A. et al. Centriolar CPAP/SAS-4 imparts slow processive microtubule growth. Dev. Cell 37, 362–376 (2016).

Job Nr	1	2	3	4
Job Name	Low-zoom autofocus	Low-zoom population image	High-zoom autofocus	High-zoom single-cell image
Voxel width	4 µm	0.312 µm	0.71 µm	0.228 µm
z-dimension	0.1 µm	3 µm	0.5 µm	0.75 µm
Nr. of z	300	3	61	32
xy dimensions	160 µm x 4 µm	607 µm x 607 µm	22.8 µm x 22.8 µm	22.8 µm x 22.8 µm
Pinhole size	41.5 µm	218.4 µm	42.2 µm	49.3 µm
Scan speed / Averaging	8 (bi) / 1	8 (uni) / 4 (mean)	16 (uni) / 1	10 (uni) / 2 (mean)
Pixel dwell time	2.18 µs	4.89 µs	0.76 µs	3.39 µs
Magnification	1	0,5	7,7	7,7
Laser lines (power)	640 nm (5%)	488 nm (10%), 640 (5%)	488 nm (10%)	488 nm (10%), 640 (5%)
Detection	630-700 nm	410-559 nm, 656-700 nm	410-559 nm	410-559 nm, 656-700 nm
