# Supplementary figures and images for: Cell state-specific cytoplasmic density controls spindle architecture and scaling
Source: Nat Cell Biol. 2025 Jun 13;27(6):959–71. doi: 10.1038/s41556-025-01678-x (PMC12173940; doi:10.1038/s41556-025-01678-x)

Figure 1d

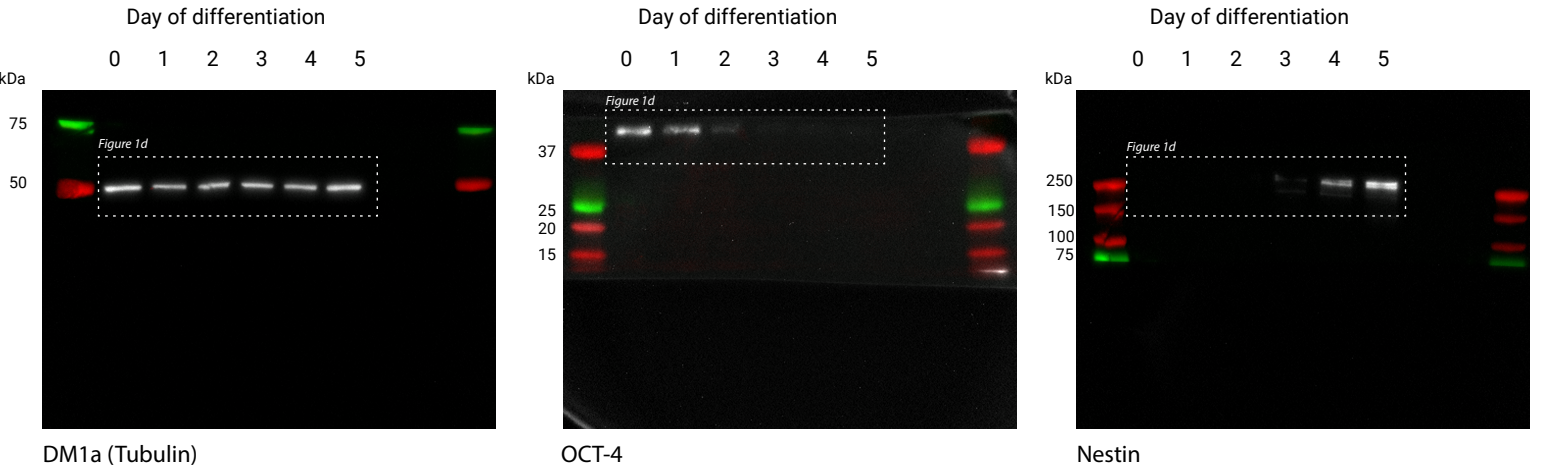

Supplement: Supplementary file 11 — Unprocessed blots. [file 41556_2025_1678_MOESM11_ESM.pdf]

Figure 3e

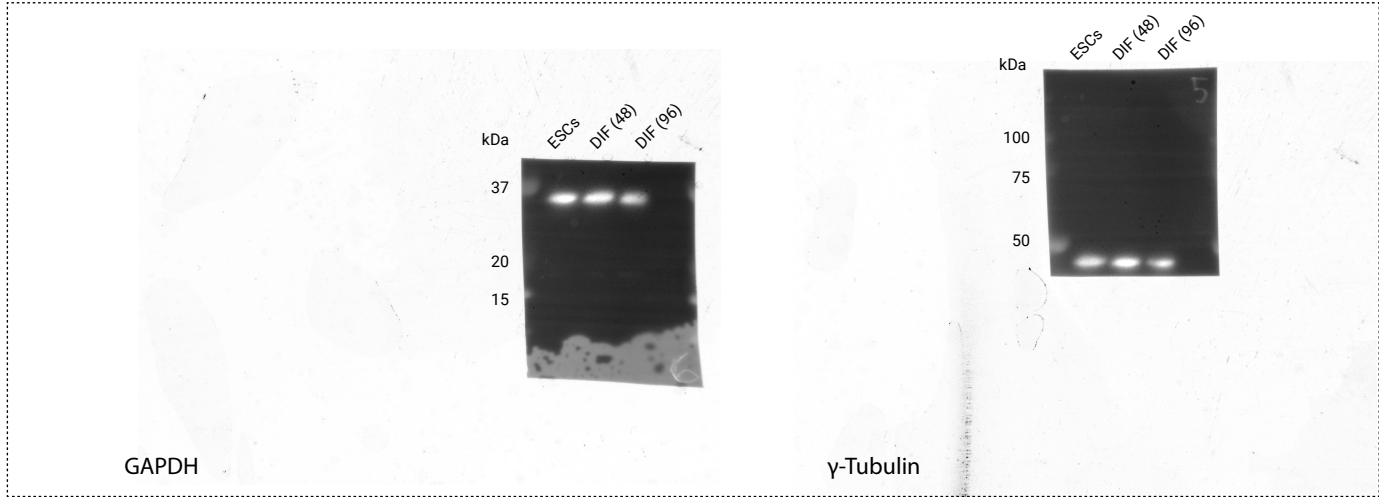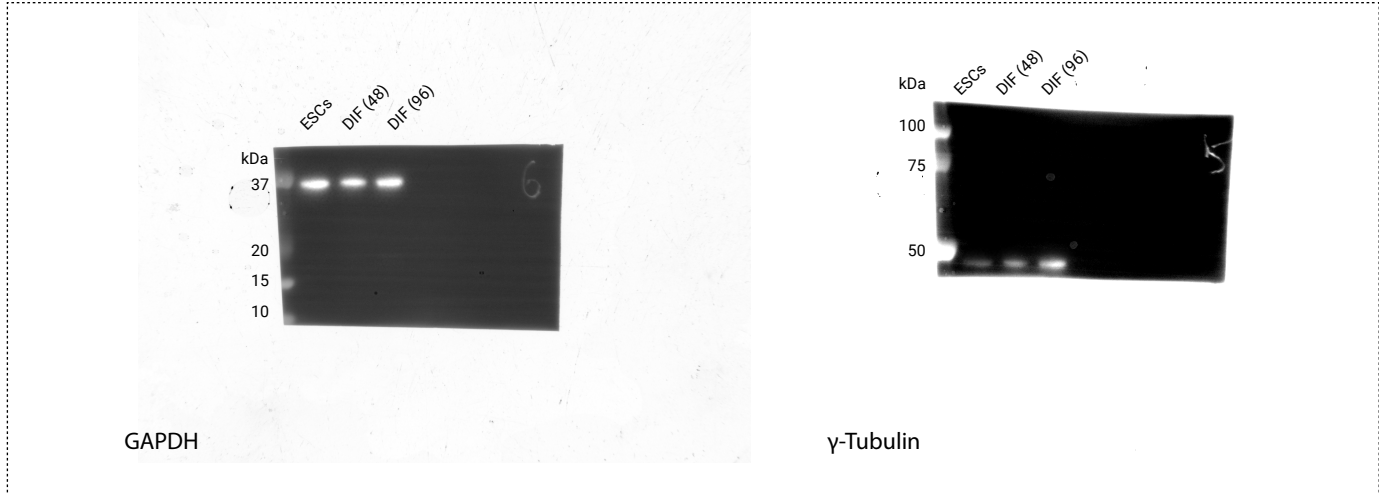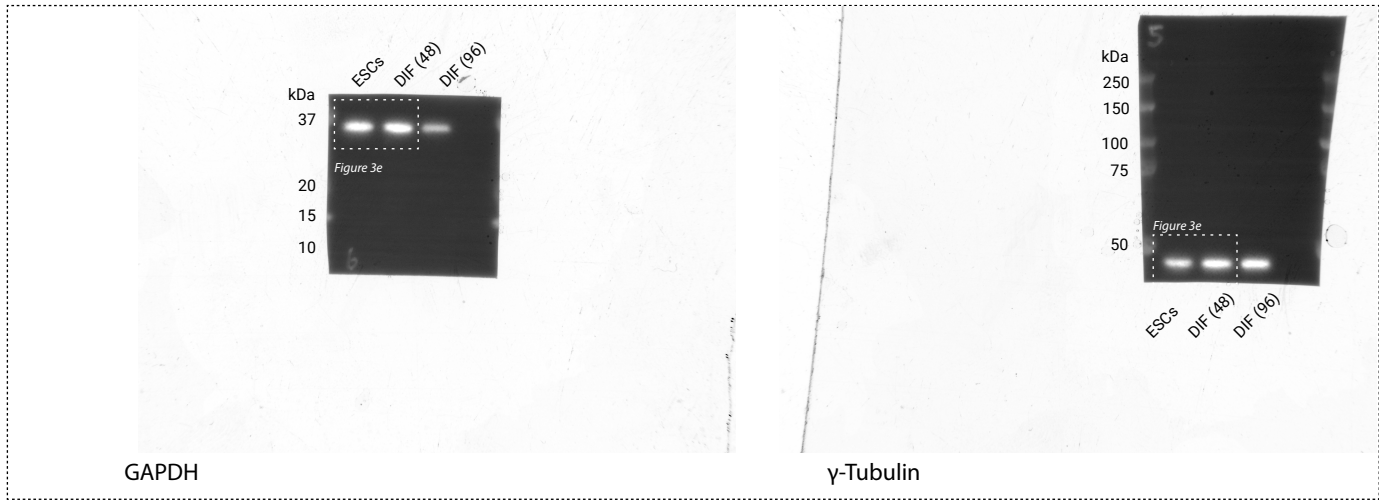

Supplement: Supplementary file 14 — Unprocessed blots. [file 41556_2025_1678_MOESM14_ESM.pdf]

Figure 4c

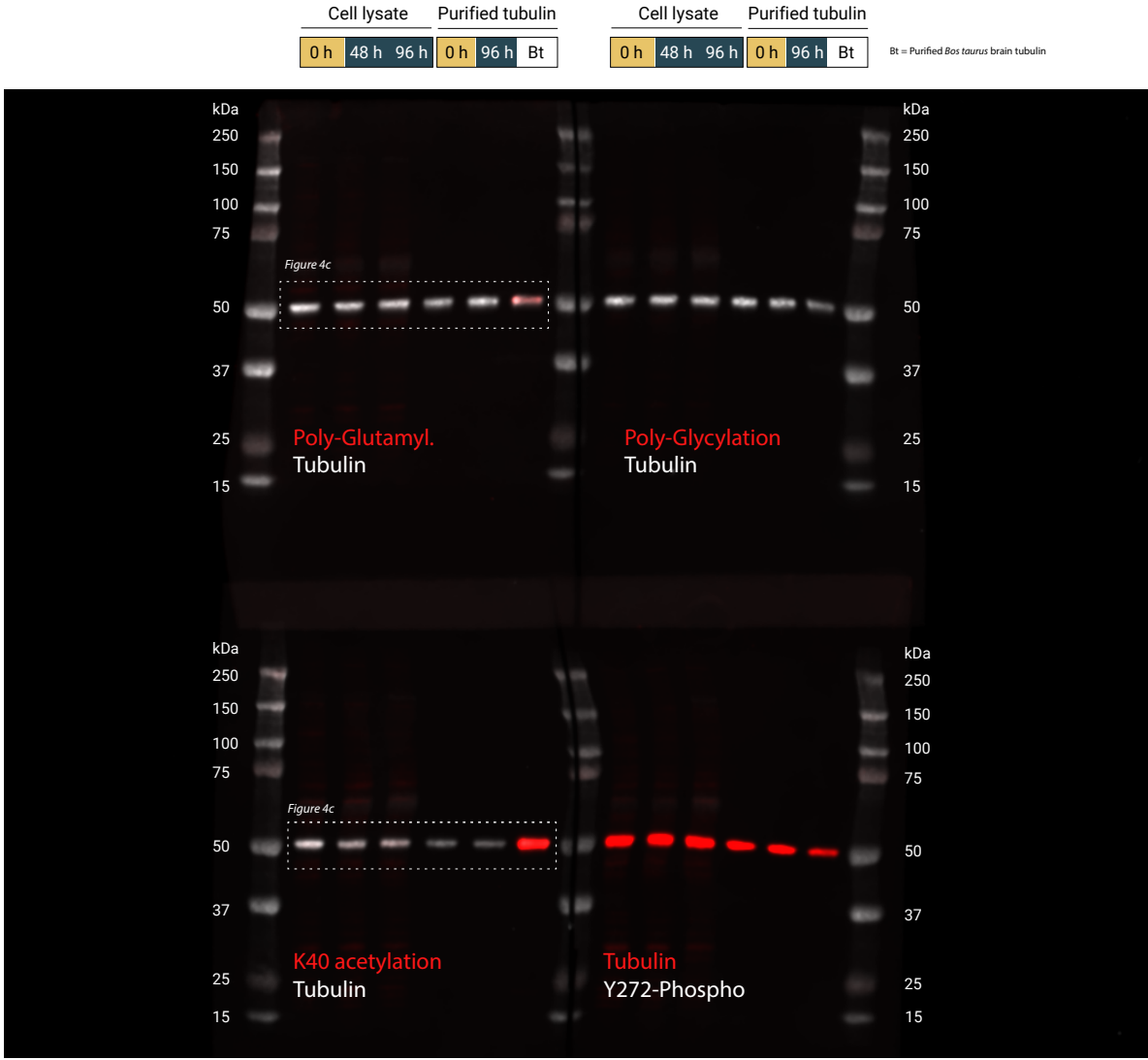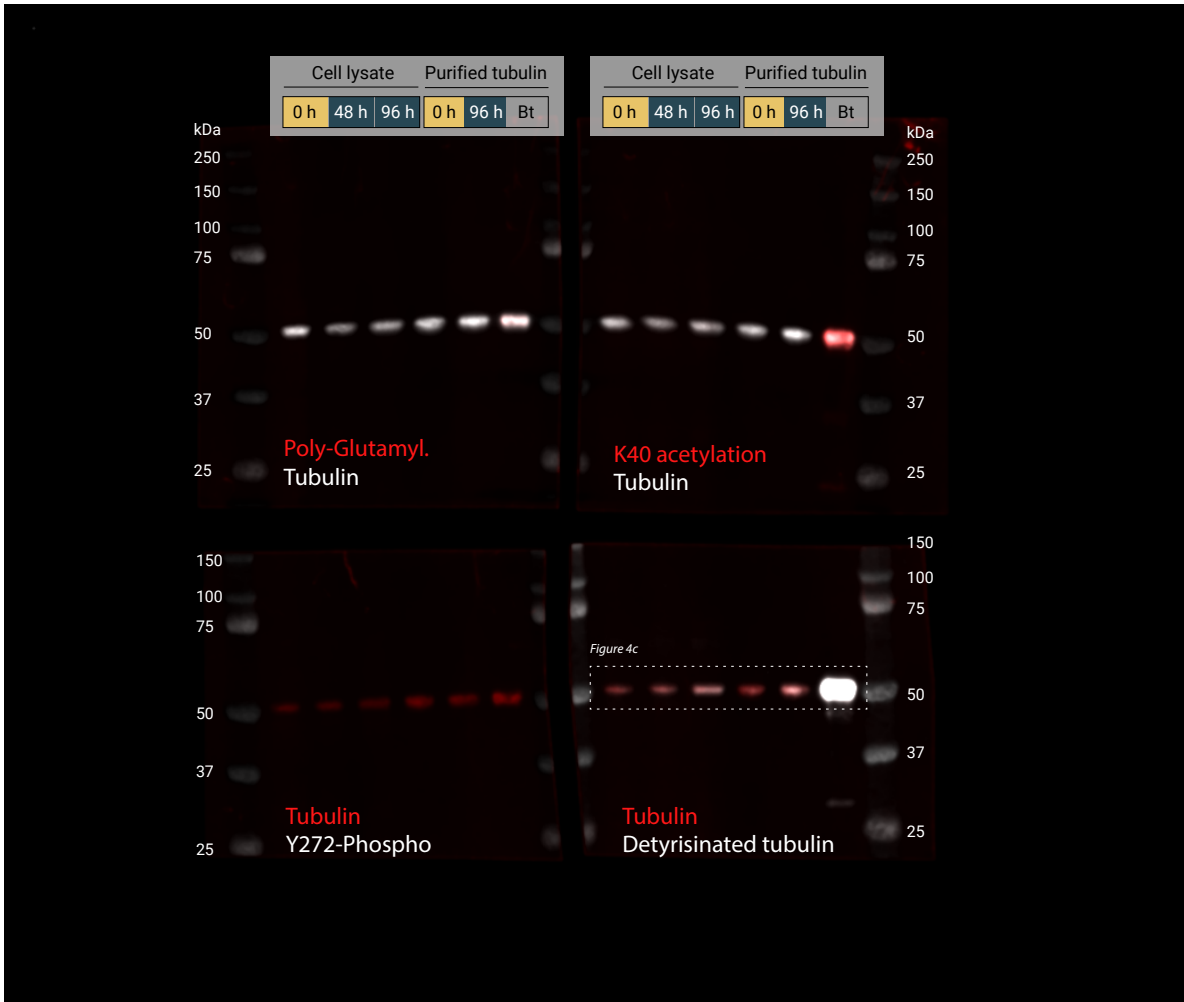

Figure 4d

Lysate batch 1

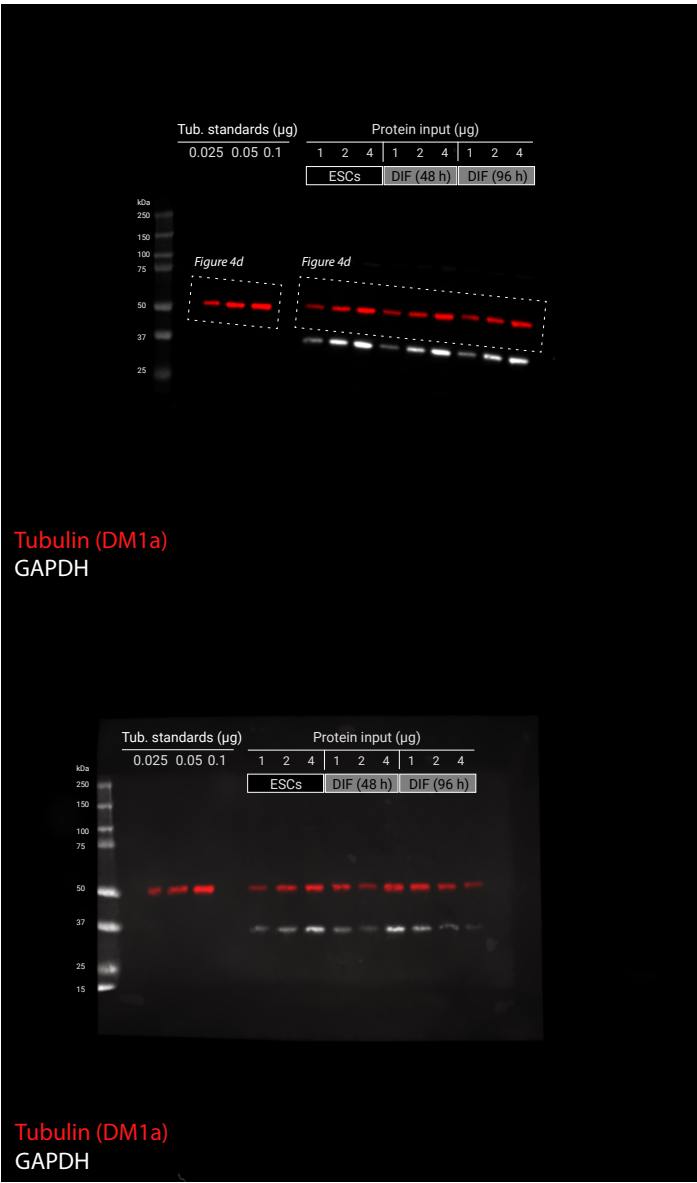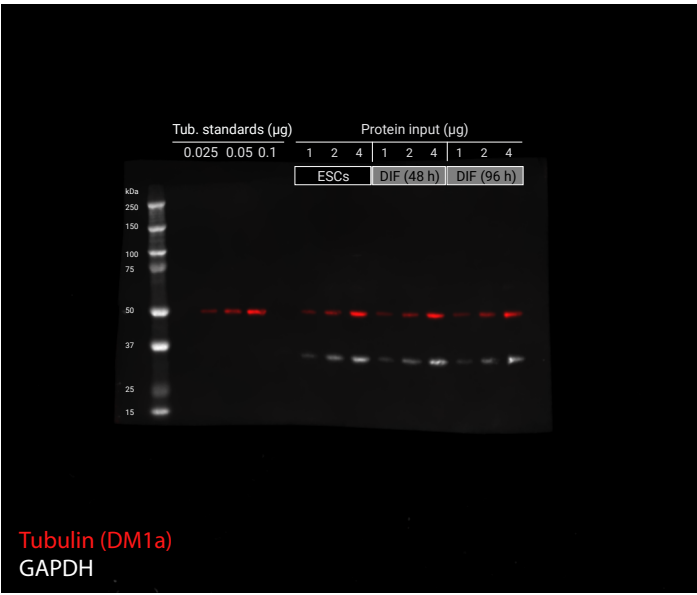

Coomassie Gel

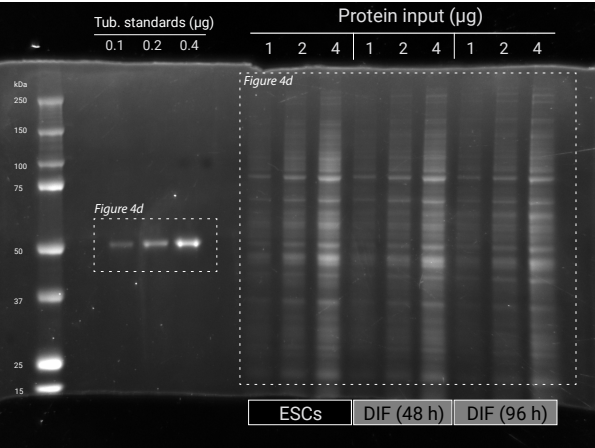

Lysate batch 2

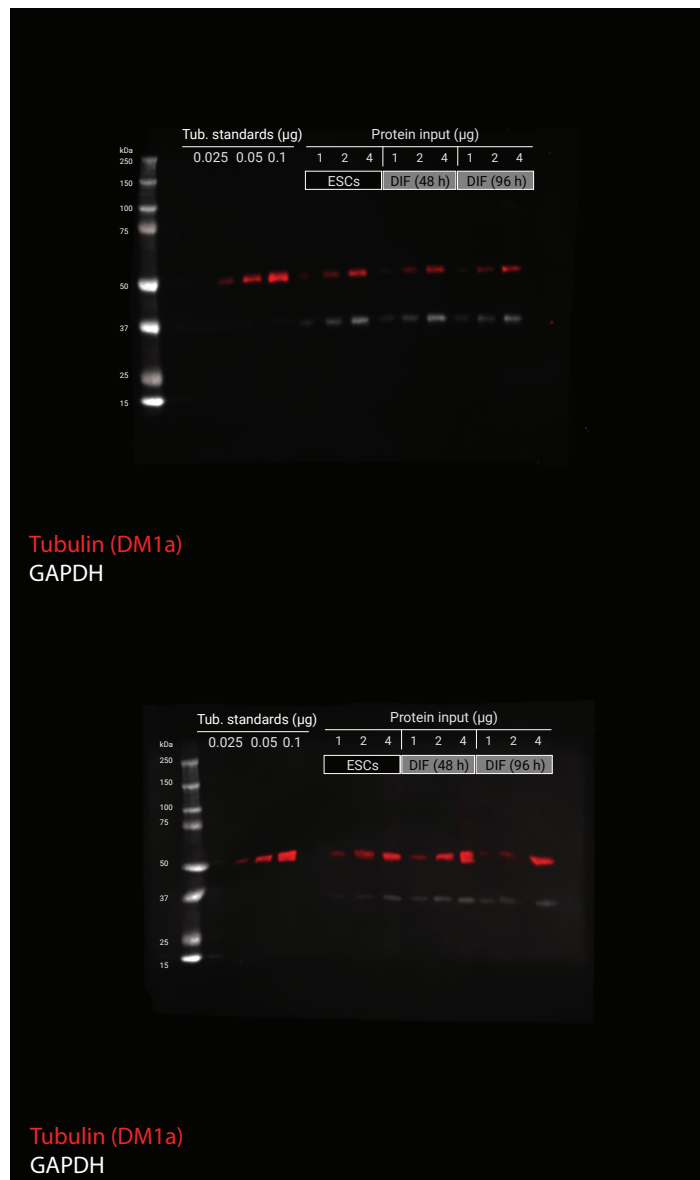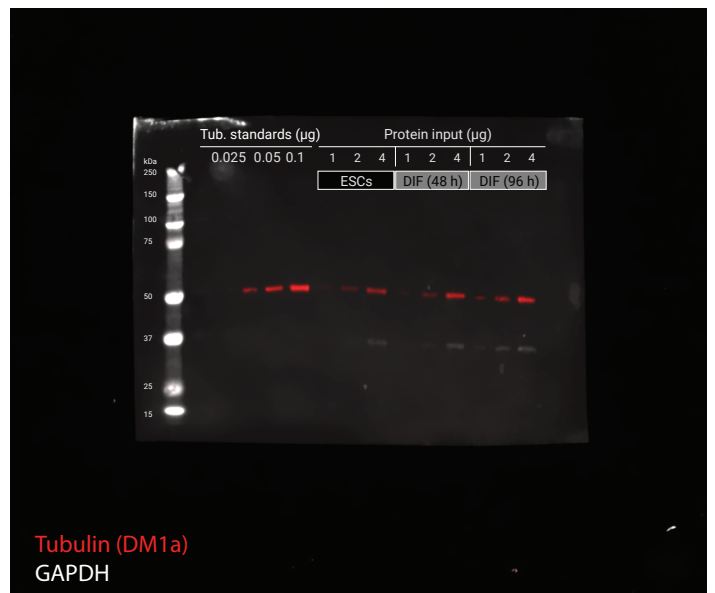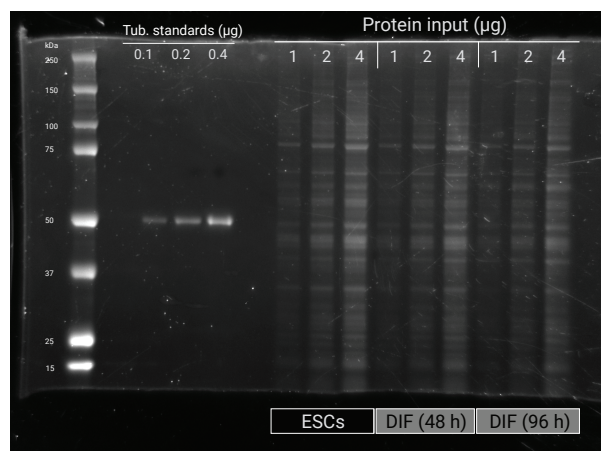

Figure 4d

Lysate batch 3

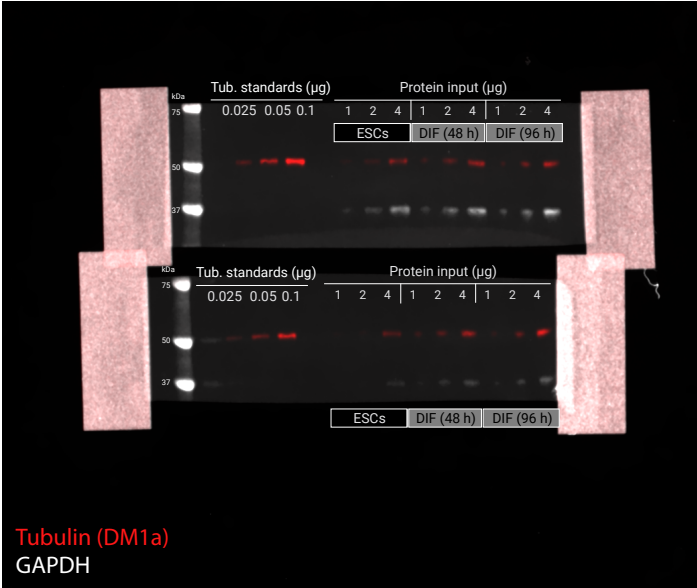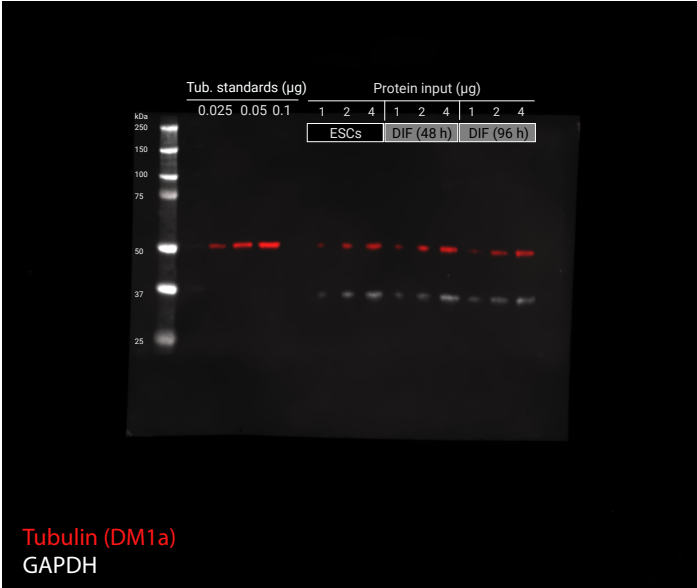

Coomassie Gel

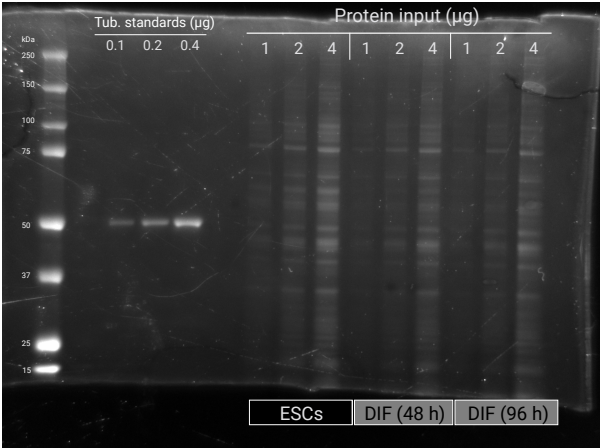

Supplement: Supplementary file 16 — Unprocessed blots. [file 41556_2025_1678_MOESM16_ESM.pdf]

Extended Data Figure 3e  
Extended Data Figure 4d  
Extended Data Figure 5b

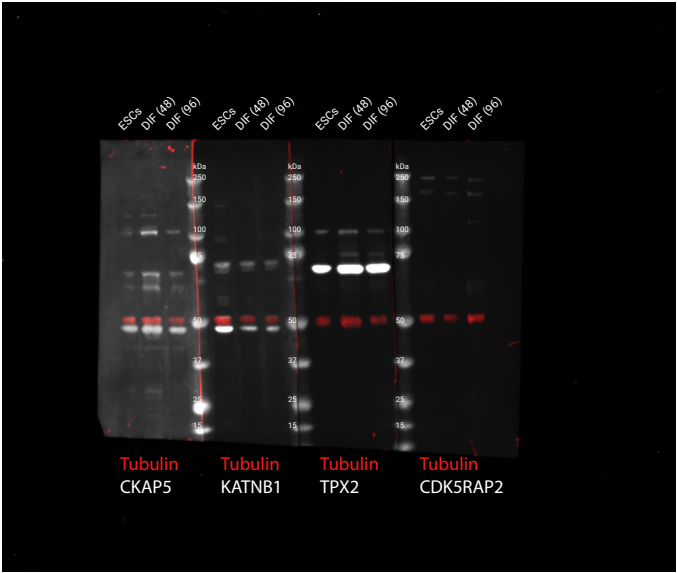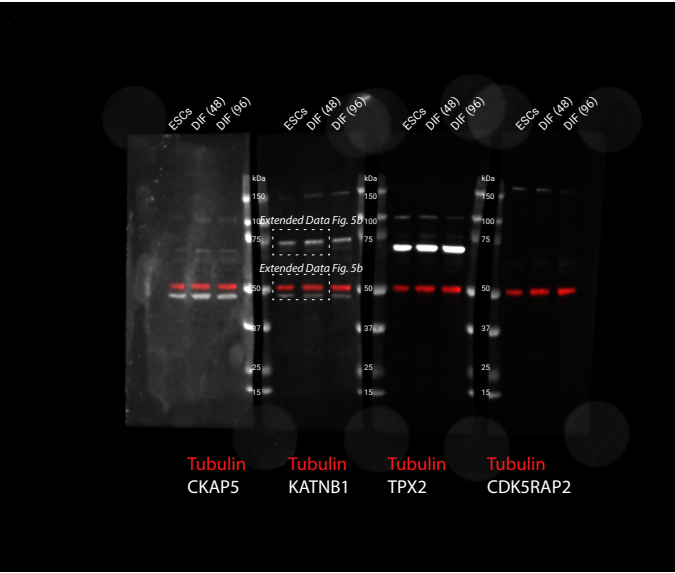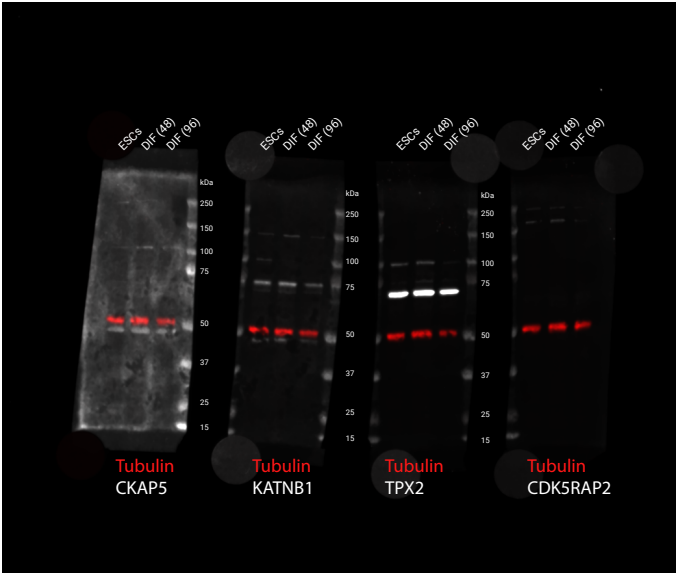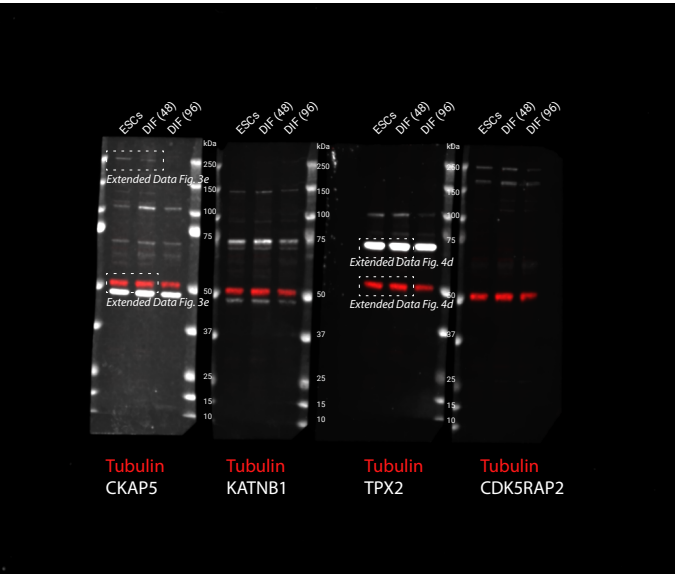

Extended Data Figure 4a

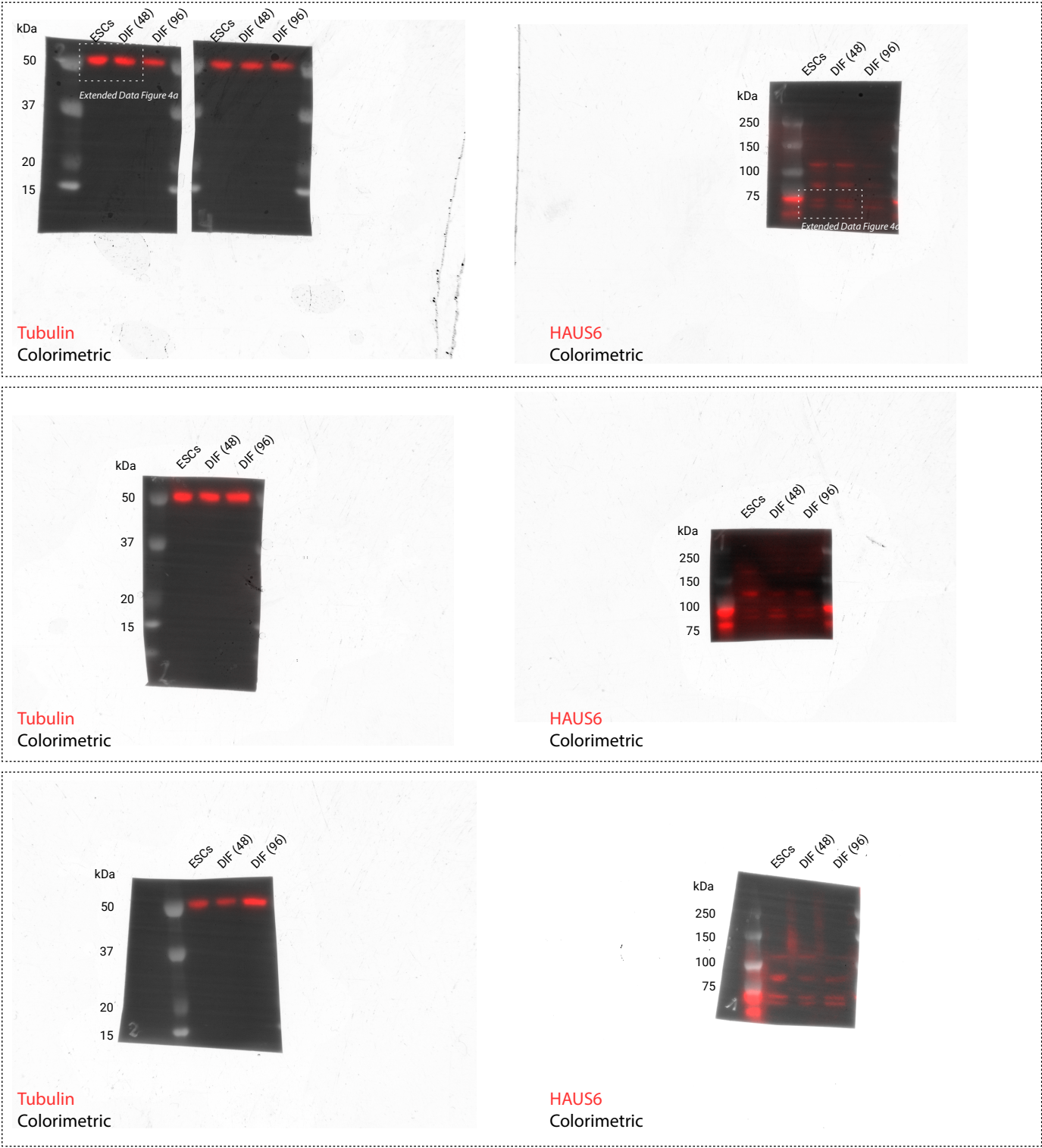

Supplement: Supplementary file 23 — Unprocessed blots. [file 41556_2025_1678_MOESM23_ESM.pdf]
