# Supplementary material for: Cell state-specific cytoplasmic density controls spindle architecture and scaling
Source: Nat Cell Biol. 2025 Jun 13;27(6):959–71. doi: 10.1038/s41556-025-01678-x (PMC12173940; doi:10.1038/s41556-025-01678-x)

Extended Data Figure 3e
Extended Data Figure 4d
Extended Data Figure 5b

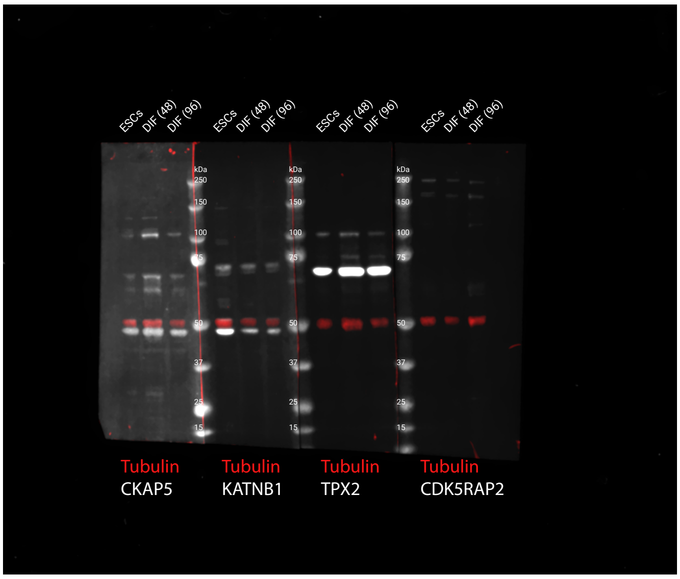

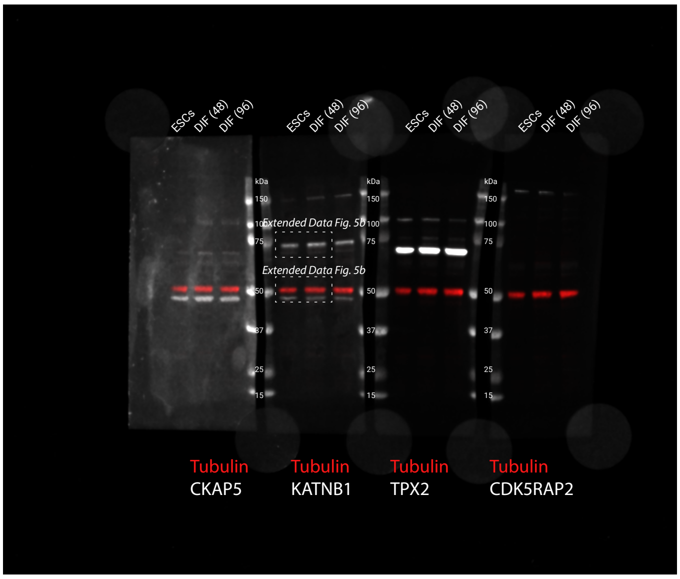

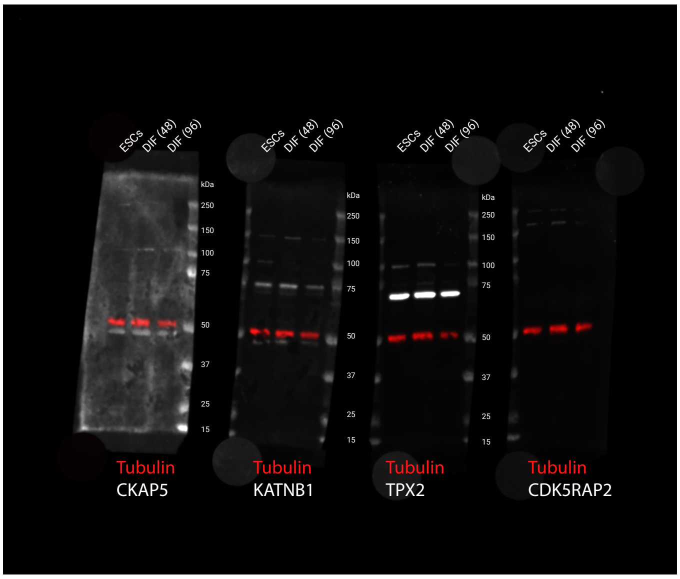

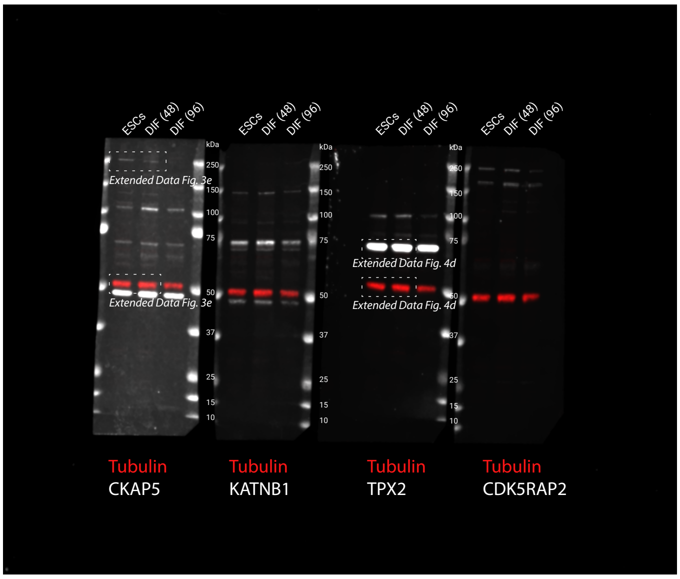

Extended Data Figure 3b
Extended Data Figure 5a

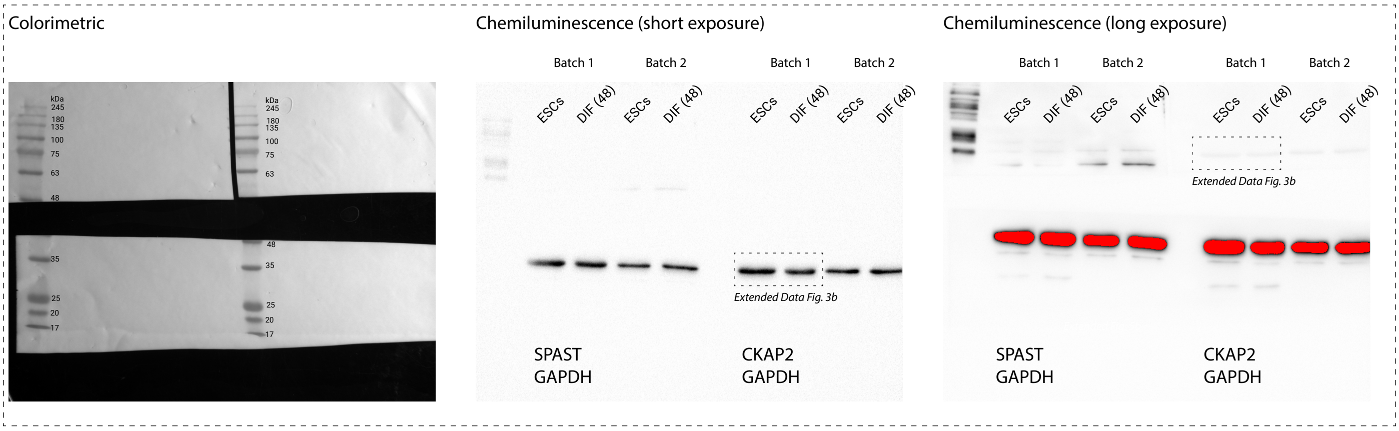

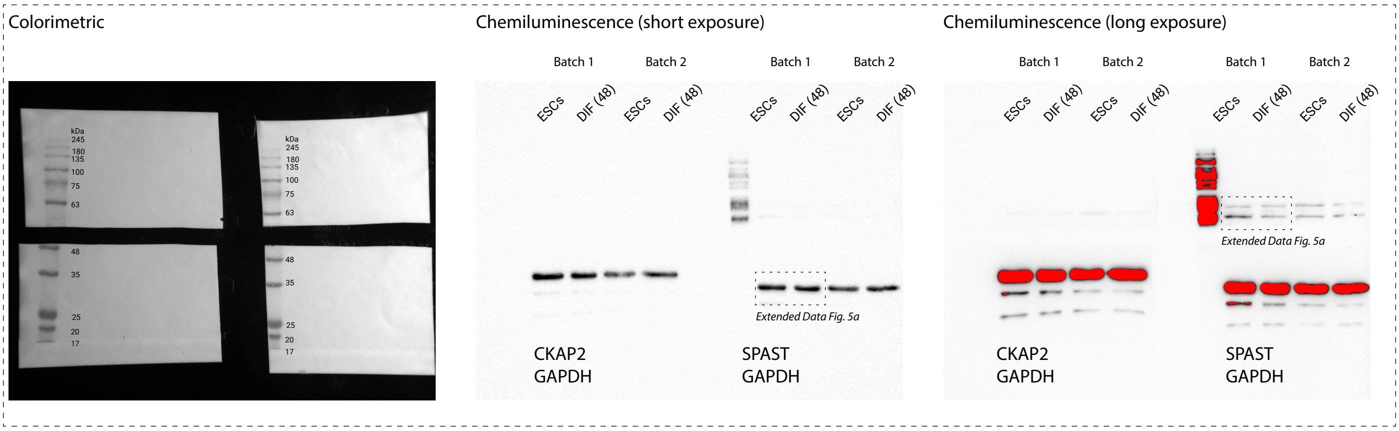

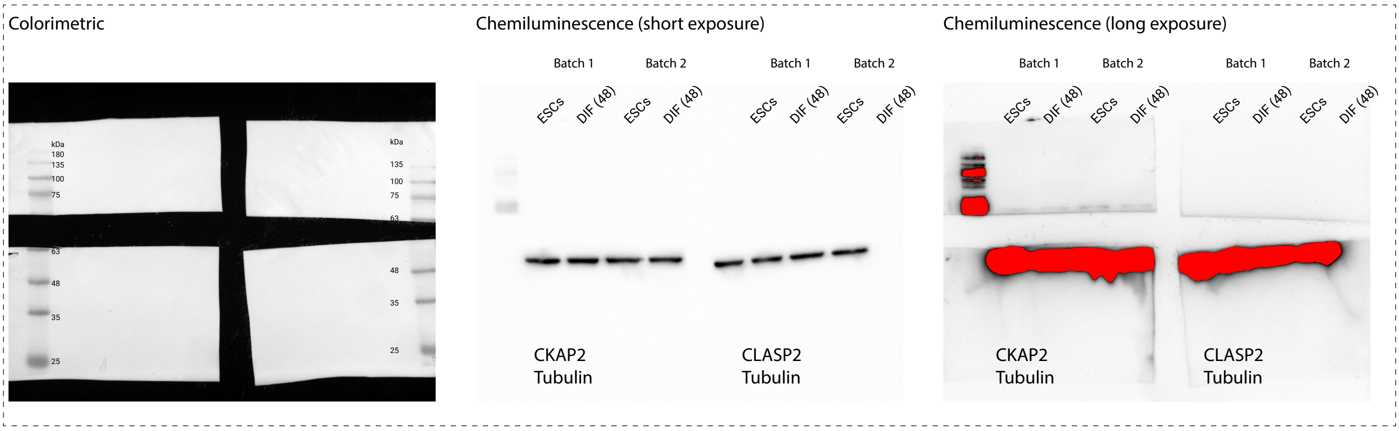

Extended Data Figure 3f

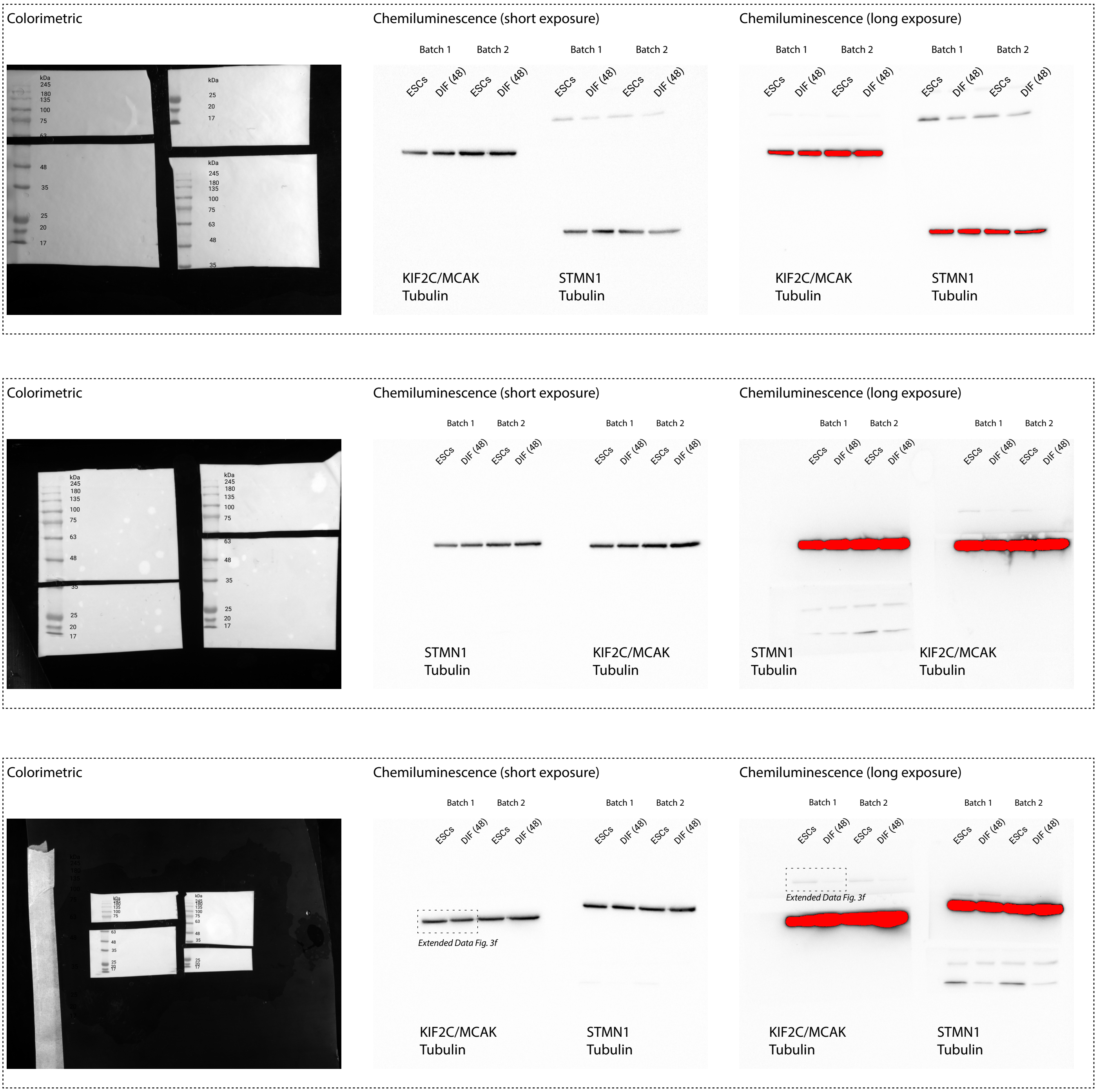

Supplement: Supplementary file 21 — Unprocessed blots. [file 41556_2025_1678_MOESM21_ESM.pdf]
