# Supplementary material for: Cell state-specific cytoplasmic density controls spindle architecture and scaling
Source: Nat Cell Biol. 2025 Jun 13;27(6):959–71. doi: 10.1038/s41556-025-01678-x (PMC12173940; doi:10.1038/s41556-025-01678-x)

Extended Data Figure 3e
Extended Data Figure 4d
Extended Data Figure 5b

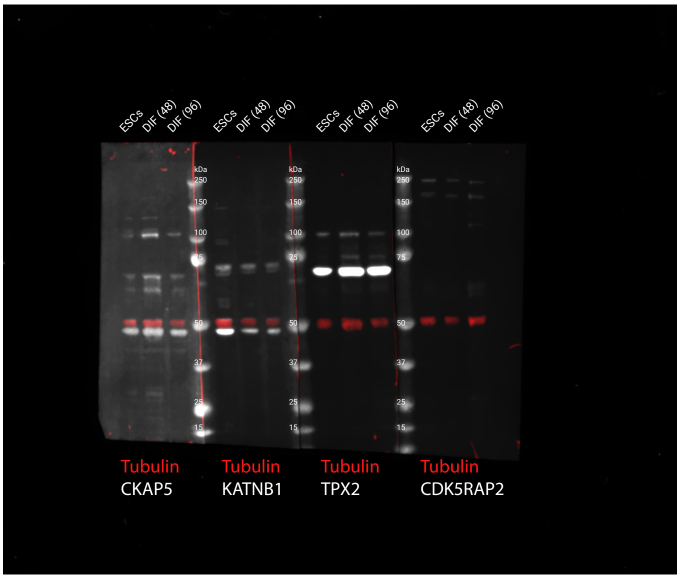

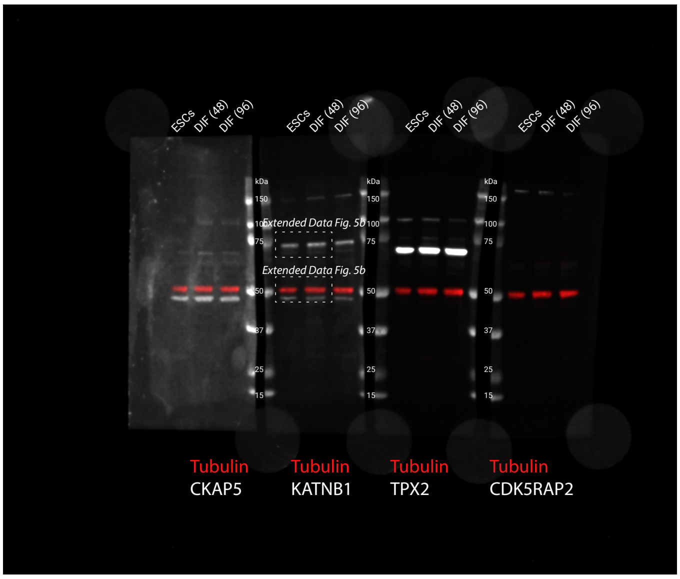

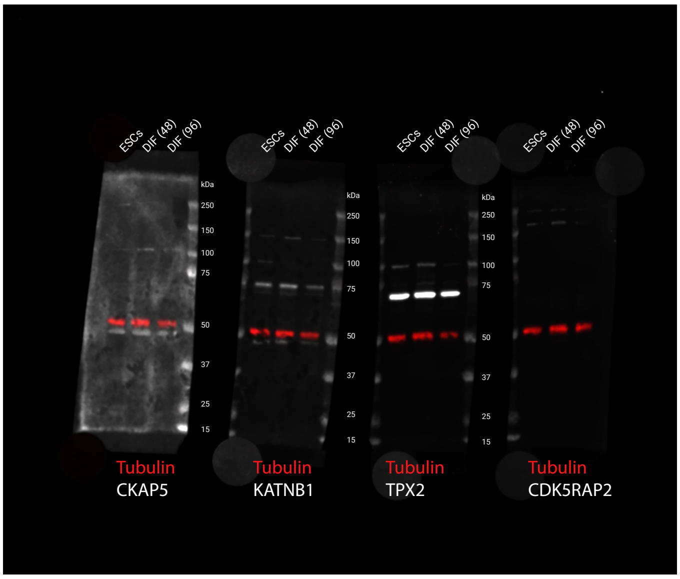

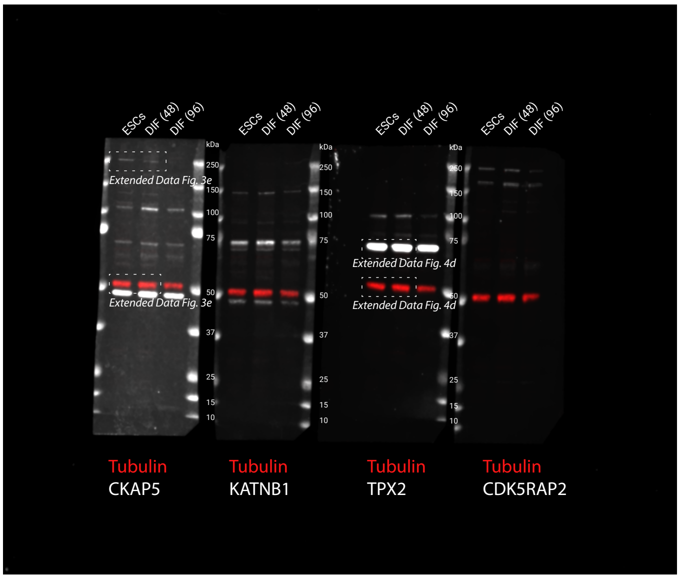

Extended Data Figure 3b
Extended Data Figure 5a

Colorimetric

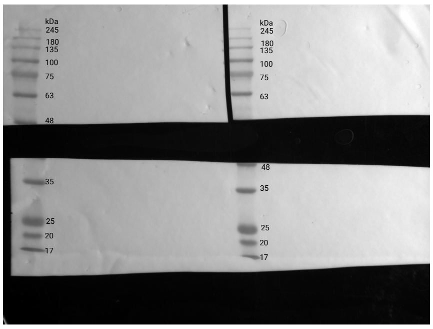

Chemiluminescence (short exposure)

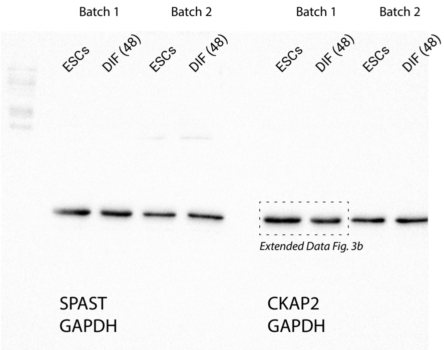

Chemiluminescence (long exposure)

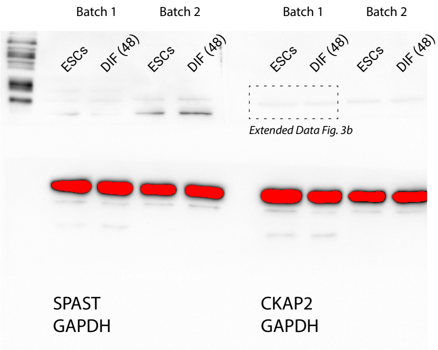

Colorimetric

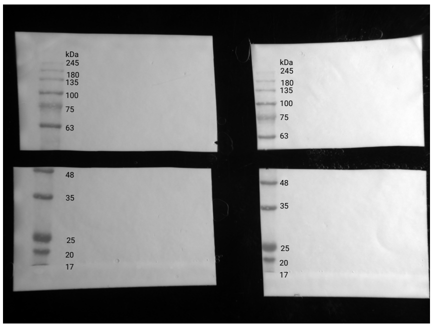

Chemiluminescence (short exposure)

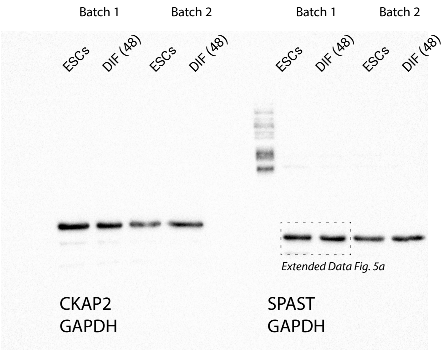

Chemiluminescence (long exposure)

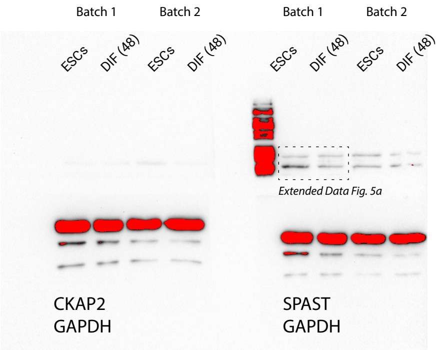

Colorimetric

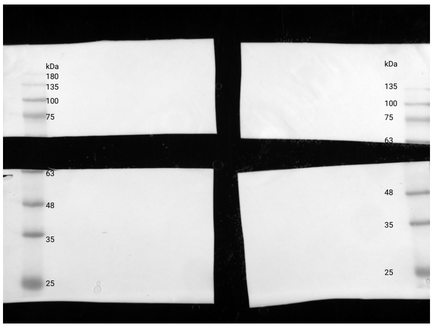

Chemiluminescence (short exposure)

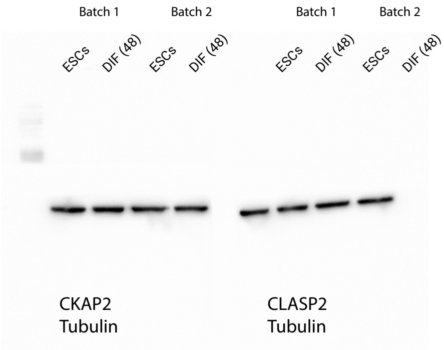

Chemiluminescence (long exposure)

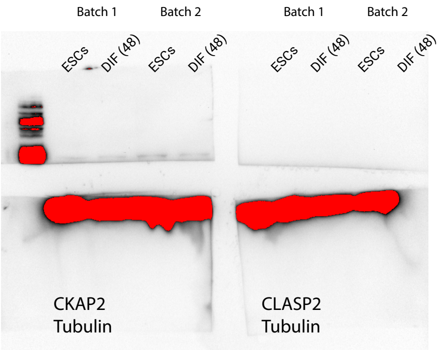

Extended Data Figure 5h

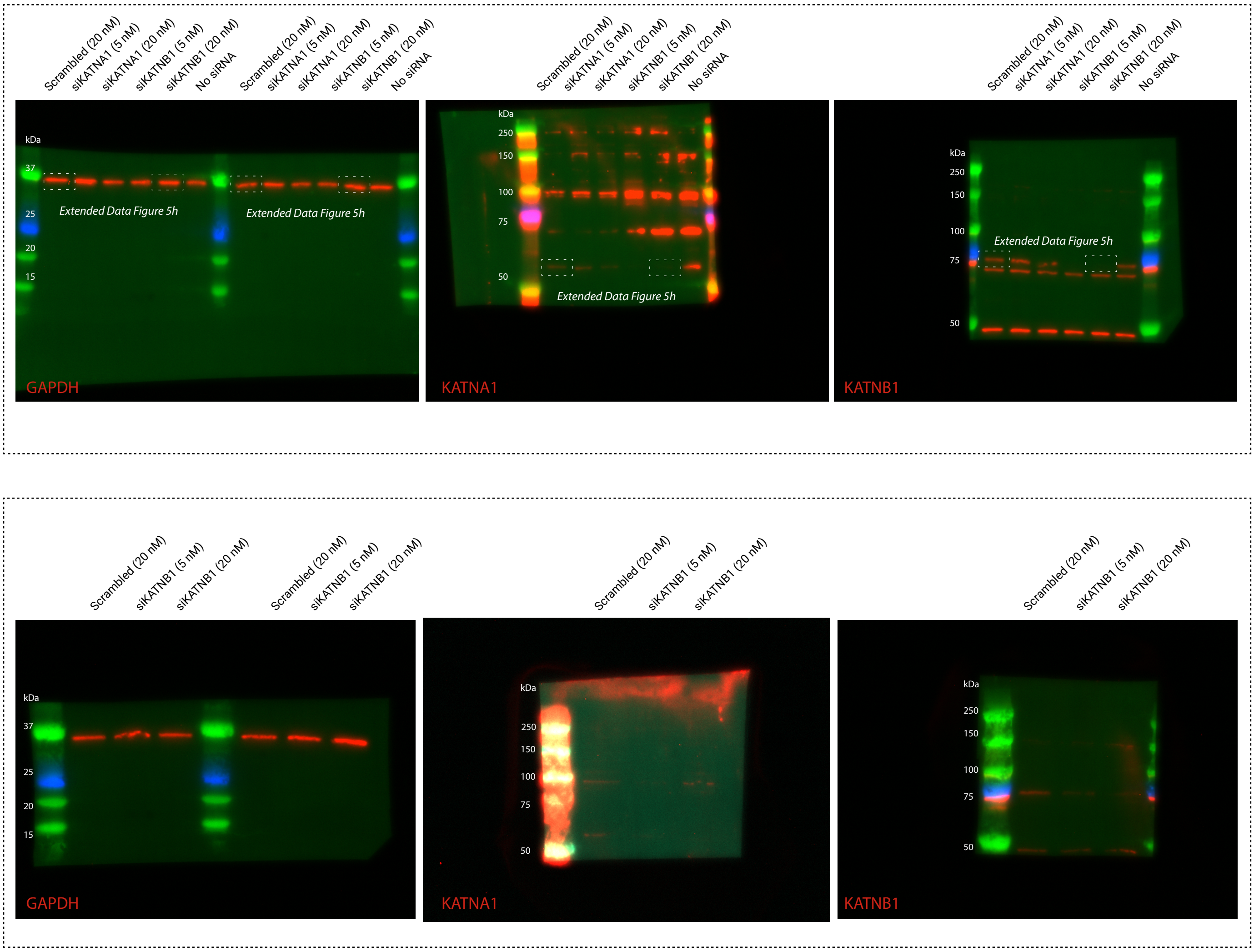

Supplement: Supplementary file 25 — Unprocessed blots. [file 41556_2025_1678_MOESM25_ESM.pdf]
